# Supplementary material for: Histone proteoform analysis reveals epigenetic changes in adult mouse brown adipose tissue in response to cold stress
Source: bioRxiv. 2024 Jan 22:2023.07.30.551059. Preprint. [Version 2] doi: 10.1101/2023.07.30.551059 (PMC10849524; doi:10.1101/2023.07.30.551059)
Supplement: Supplement 1 — Table S1. Average body weights, fat mass, lean mass, tissue weights, and nonfasting glucose of male C57BL/6J mice after exposure to thermoneutral or chronic cold housing temperatures. Results shown are mean α SEM. *p <0.05 for SC vs TN, #p <0.05 for SC vs RT and TN, and @p <0.05 for RT vs TN. TN: thermoneutral, RT: room temperature, SC: severe cold. [file media-1.pdf]

Table S1. Average body weights, fat mass, lean mass, tissue weights, and nonfasting glucose of male C57BL/6J mice after exposure to thermoneutral or chronic cold housing temperatures. Results shown are mean ± SEM. *p <0.05 for SC vs TN, $^{\#}p$ <0.05 for SC vs RT, and $^{@}p$ <0.05 for RT vs TN. TN: thermoneutral, RT: room temperature, SC: severe cold.

	**TN (28°C)**	**RT (22°C)**	**SC (8°C)**
**Body Weight (g)**	27.23 ± 0.39	28.03 ± 0.57	25.58 ± 0.46$^{*\#}$
**Fat Mass (g)**	3.73 ± 0.27	3.41 ± 0.39	2.61 ± 0.14*
**Lean Mass (g)**	22.13 ± 0.29	23.19 ± 0.55	21.69 ± 0.46
**BAT Weight (g)**	0.1086 ± 0.0046	0.1030 ± 0.0070	0.1051 ± 0.0034
**gWAT Weight (g)**	0.781 ± 0.053	0.673 ± 0.047	0.444 ± 0.021$^{*\#}$
**Kidney Weight (g)**	0.1651 ± 0.0054	0.1898 ± 0.0060$^{@}$	0.2014 ± 0.0063*
**Liver Weight (g)**	1.458 ± 0.040	1.535 ± 0.062	1.416 ± 0.048
**Spleen Weight (g)**	0.0758 ± 0.0033	0.0959 ± 0.0088	0.0791 ± 0.0045
**Nonfasting Glucose (mg/dl)**	180 ± 8	197 ± 9	192 ± 9
