# Supplementary material for: Histone proteoform analysis reveals epigenetic changes in adult mouse brown adipose tissue in response to cold stress
Source: bioRxiv. 2024 Jan 22:2023.07.30.551059. Preprint. [Version 2] doi: 10.1101/2023.07.30.551059 (PMC10849524; doi:10.1101/2023.07.30.551059)
Supplement: Supplement 2 — Table S2. Results of method optimization of histone isolation from BAT. Color scheme indicates the highest yield of histones in green (2) and the lowest yield of histones in red. Mass histone is calculated using offline HPLC peak area and standard curve. [file media-2.pdf]

Table S2. *Results of method optimization of histone isolation from BAT.* Color scheme indicates the highest yield of histones in green (2) and the lowest yield of histones in red. Mass histone is calculated using offline HPLC peak area and standard curve.

	**NP-40 %**	**NIB-only washes**	**NI spins**	**AE apins**	**g BAT**	**µg H4/ g BAT**	**µg H3.2/ g BAT**
1	0.3	3	10 minutes	10 minutes	0.1111	11.57	14.28
2	0.3	3	10 minutes	5 minutes	0.1019	108.82	74.22
3	0.3	3	10 minutes	5 minutes	0.1030	24.21	15.71
4	0.5	3	10 minutes	5 minutes	0.1615	45.35	25.07
5	1	3	10 minutes	5 minutes	0.1170	16.33	8.97
6	0.3	3	10 minutes	5 minutes	0.0815	32.20	15.23
7	0.5	3	10 minutes	5 minutes	0.0513	61.34	27.66
8	1	3	10 minutes	5 minutes	0.0607	23.34	8.96
