# Supplementary material for: Histone proteoform analysis reveals epigenetic changes in adult mouse brown adipose tissue in response to cold stress
Source: bioRxiv. 2024 Jan 22:2023.07.30.551059. Preprint. [Version 2] doi: 10.1101/2023.07.30.551059 (PMC10849524; doi:10.1101/2023.07.30.551059)
Supplement: Supplement 3 — Table S3. Mass of histone H3.2 and H4 obtained from (a) BAT and (b) liver at different housing temperatures. Mass histone is calculated using offline HPLC peak area and standard curve. TN: thermoneutral; RT: room temperature; SC: severe cold. [file media-3.pdf]

Table S3. *Mass of histone H3.2 and H4 obtained from* ***(a)*** *BAT and* ***(b)*** *liver at different housing temperatures.* Mass histone is calculated using offline HPLC peak area and standard curve. TN: thermoneutral; RT: room temperature; SC: severe cold.

	Housing temp.	µg H3.2/ g tissue	µg H4/ g tissue
A) BAT	SC	37 ± 4	54 ± 8
	RT	33 ± 6	46 ± 7
	TN	30 ± 10	36 ± 11
	Average	33 ± 7	45 ± 9
B) Liver	SC	18 ± 2	27 ± 5
	RT	14 ± 3	18 ± 2
	TN	15 ± 2	19 ± 3
	Average	16 ± 2	21 ± 3
